# Adaptation of the Grasha Riechman Student Learning Style Survey and Teaching Style Inventory to assess individual teaching and learning styles in a quality improvement collaborative

**DOI:** 10.1186/s12909-016-0772-4

**Published:** 2016-09-29

**Authors:** James H. Ford, James M. Robinson, Meg E. Wise

**Affiliations:** 1 Center for Health Systems Research and Analysis, University of Wisconsin-Madison, 610 Walnut Street, Madison, WI 53726 USA; 2 Sonderegger Research Center, School of Pharmacy, University of Wisconsin-Madison, Madison, WI 53705 USA

**Keywords:** Quality improvement collaborative, Teaching style, Learning style, Coaches, Change leaders, Executive sponsors, Principal components analysis, NIATx

## Abstract

**Background:**

NIATx200, a quality improvement collaborative, involved 201 substance abuse clinics. Each clinic was randomized to one of four implementation strategies: (a) interest circle calls, (b) learning sessions, (c) coach only or (d) a combination of all three. Each strategy was led by NIATx200 coaches who provided direct coaching or facilitated the interest circle and learning session interventions.

**Methods:**

Eligibility was limited to NIATx200 coaches (*N* = 18), and the executive sponsor/change leader of participating clinics (*N* = 389). Participants were invited to complete a modified Grasha Riechmann Student Learning Style Survey and Teaching Style Inventory. Principal components analysis determined participants’ preferred learning and teaching styles.

**Results:**

Responses were received from 17 (94.4 %) of the coaches. Seventy-two individuals were excluded from the initial sample of change leaders and executive sponsors (*N* = 389). Responses were received from 80 persons (25.2 %) of the contactable individuals. Six learning profiles for the executive sponsors and change leaders were identified: Collaborative/Competitive (*N* = 28, 36.4 %); Collaborative/Participatory (*N* = 19, 24.7 %); Collaborative only (*N* = 17, 22.1 %); Collaborative/Dependent (*N* = 6, 7.8 %); Independent (*N* = 3, 5.2 %); and Avoidant/Dependent (*N* = 3, 3.9 %). NIATx200 coaches relied primarily on one of four coaching profiles: Facilitator (*N* = 7, 41.2 %), Facilitator/Delegator (*N* = 6, 35.3 %), Facilitator/Personal Model (*N* = 3, 17.6 %) and Delegator (*N* = 1, 5.9 %). Coaches also supported their primary coaching profiles with one of eight different secondary coaching profiles.

**Conclusions:**

The study is one of the first to assess teaching and learning styles within a QIC. Results indicate that individual learners (change leaders and executive sponsors) and coaches utilize multiple approaches in the teaching and practice-based learning of quality improvement (QI) processes. Identification teaching profiles could be used to tailor the collaborative structure and content delivery. Efforts to accommodate learning styles would facilitate knowledge acquisition enhancing the effectiveness of a QI collaborative to improve organizational processes and outcomes.

**Trial registration:**

ClinicalTrials.gov Identifier: NCT00934141 Registered July 6, 2009. Retrospectively registered.

**Electronic supplementary material:**

The online version of this article (doi:10.1186/s12909-016-0772-4) contains supplementary material, which is available to authorized users.

## Background

Quality improvement collaboratives (QIC) are widely used in healthcare organizations to improve organizational processes and client outcomes [1–5]. Such collaboratives include multiple approaches to teaching quality improvement to enhance participants’ knowledge, understanding and skills. A coach led QIC enlists a “coach” (teacher) to engage key people in an organization (the change team, or learners) to design, implement and sustain new processes that improve the delivery of their services [3, 5–14]. In that capacity, the coach employs multiple strategies (e.g., site visits or monthly calls) to help the change team build their skills in applying evidence-based QI tools and techniques to their unique circumstances. How the coach teaches and the change team learns—and how these work together—are the salient components in a QIC. Coaches like other teachers tailor the learning interchange to individual needs and goals and use active (e.g., a hands-on Plan-Do-Study-Act activity) vs. passive (e.g., a lecture on data collection and measurement) learning approaches to develop skills and build confidence [15–17]. The change team members must then learn to apply the skills taught. Successful learning is thus directly impacted by the quality of coaching. However, research has not assessed how a coaches’ teaching style or the change leader’s learning style impacts the outcome in a QIC.

Applying both received and practice-based knowledge, versus simply acquiring knowledge, represents higher-order learning. Thus the educator’s task is to account for an individual’s learning style in a way that effectively motivates this higher-order learning process [18–21]. Medical education, where practicing physicians mentor residents, has long used this teaching and learning model [22–27]. The NIATx Change Leader Academy adapted this model by providing active learning opportunities for clinical and administrative staff to become effective change leaders in their organizations [28, 29]. The change leader’s learning style and the coaches teaching style therefore are important considerations in a coach-facilitated QIC. Yet the research and practice of teaching and learning styles are focused on traditional educational settings.

The Grasha-Riechmann model integrates individual teaching and learning style and demonstrates how the stylistic qualities of teachers and students can enhance the nature and quality of the learning experience While most teachers have a preferred teaching style, they use a mix of styles to engage a wide array of learners who might prefer interactive, experiential or didactic teaching approaches [30–32]. The use of different approaches also accommodates individual preferences for different learning modalities-visual, auditory, kinesthetic or tactile [33]. For example, material might be taught through a lecture using slides which is then supported by an interactive exercise involving a small group discussion of a case study with questions or an experiential hands-on learning opportunity to convey knowledge or skills. Educators who understand their preferred teaching style(s) are better able to identify and enlist a variety of teaching strategies, as needed [34]. Grasha-Reichmann identified five teaching (Delegator, Expert, Facilitator, Formal Authority and Personal Model) styles utilized within an educational environment.

Coffield and colleagues [35] classified the Grasha Reichmann model as an approach that focused on how personal attributes (e.g., motivation) influence learning strategies, approaches and concepts associated with learning. The Grasha-Riechmann Student Learning Style Survey (GRSLSS) has been studied across a variety of educational settings [18, 21, 30, 36–43]. The survey identifies the degree to which an individual prefers each of six learning styles: (a) competitive, (b) collaborative, (c) avoidant, (d) participant, (e) dependent and (f) independent [34]. The surveys utilized a five and seven point Likert scale respectively. Utilization of these integrated styles led to the clustering of different teaching profiles (e.g., Facilitator/Personal Model/Expert) and learning profiles (e.g., Collaborative/Participant/Independent) and the identification of particular teaching methods (e.g., guided readings, role play) could be utilized to increase knowledge transfer [34].

We utilized the Grasha-Riechmann integrated model because the learning delivery components of the NIATx200 QIC mirrored similar educational settings. The present study is nested within NIATx200, an 18-month cluster-randomized trial that was conducted in 201-addiction treatment clinics in five U.S. states [4]. Within the context of a quality improvement collaborative, participating clinics were assigned to one of four interventions facilitated by NIATx200 coaches: (a) interest circle calls, (b) learning sessions, (c) coach only or (d) a combination of all three.

The NIATx200 study involved three key players: process improvement coach, the change leader and the executive sponsor [44]. Each provider identified a change leader and executive sponsor. The change leader and executive sponsors represented the individual learners in the NIATx200 quality improvement collaborative. Within the context of the NIATx model, they fulfilled their roles and responsibilities [44] and more importantly, they interacted with their coach and utilized their individual process of knowledge acquisition (i.e., their preferred learning profile) to translate knowledge into action.

The coach served as the outside expert who provided knowledge about the NIATx model and quality improvement skills. In this teaching capacity, a coach could be involved in multiple interventions. Interest circle calls facilitated by either one or two coaches involved a combination of peer to peer sharing and didactic learning opportunities. A core faculty of three coaches used a curriculum roadmap to plan and deliver content in three learning sessions per state. The coach-only intervention launched with a 1-day, in-person site visit and monthly 1-h coaching calls to support the change leader in the implementation of changes to improve the study outcomes.

The learning sessions involve multiple individuals from different organizations is similar to an educational lecture class who were taught content through didactic lectures and small group breakout sessions. Interest circles divide the QIC participants into smaller groups. In these groups, content was delivered telephonically and the sessions were moderated by an assigned coach. One-on-one coaching is similar to a teacher having office hours for their students. Within the QIC, the coach worked directly with their assigned providers using in-person and monthly phone calls to deliver content. As compared to higher education’s focus on the student’s acquisition and analysis of information, coaches in QICs encourage learners to develop their capacity to lead teams that identify and solve real-life problems in complex organizations. The coaches’s teaching styles and the learning styles of the change leaders and executive sponsors, thus, may be shed light on an organization’s quality improvement outcomes.

The NIATx200 implementation period lasted 18-months. Each 6 month period focused on the implementation of Plan-Do-Study-Act rapid change cycles to improve one of the three primary outcomes: (a) decrease wait time from 1st contact to the 1st treatment session; (b) increase client retention, the percent of clients attending the 1st treatment session that reached the 4th session; and (c) improve the number of admissions. Results from the NIATx200 study found that the learning sessions, coaching and combined interventions resulted in significant wait time improvements; coach only or combination arms increased admissions and none of the interventions improved client retention [45].

How a coach teaches and the recipient learns are critical to the teaching and learning relationship in a QIC, but have not been studied or mapped to QIC outcomes. Within the context of the NIATx200 study setting, the present study adapted the Grasha-Riechmann Student Learning Style Survey (GRSLSS) and the Teaching Style Inventory (TSI) and administered these modified scales to identify retrospectively the learning styles of the change team (change leaders and executive sponsors) and the teaching styles of coaches who participated in NIATx200, a QIC involving 201 substance abuse treatment clinics. This paper answers the following research question: What is the learning and teaching styles typology in a quality improvement collaborative?

## Methods and design

### Survey modification and validation

Questions in the Grasha-Riechmann Student Learning Style Survey (GRSLSS) and the Teaching Style Inventory (TSI) were reviewed and the language modified for applicability and use within QIC (see Additional file 1 for original style definitions). Modifications to the teaching style inventory included replacing words like teaching with coaching; adding modifiers like quality improvement or using providers or change leaders instead of students. For example, one question in the original teaching style inventory read “Sharing my knowledge and expertise with students is very important to me” and in the modified inventory, it reads, “It is very important to share my quality improvement knowledge and expertise with the change leader/team.” Similar changes were made for the GRSLSS. For example, an original question read, “Working with other students on class activities is something I enjoy doing” and in the modified survey, it now reads, “I enjoy working with other change team members to implement quality improvement in our organization.” The modified instruments utilized the same five point ((1 = Strongly Disagree to 5 = Strongly Agree) and seven point (1 = Strongly Disagree to 7 = Strongly Agree) Likert scales as the original Learning Style Survey and Teaching Inventory respectively.

Prior to implementation, we pilot tested the revised QICTSI and QILSS with a convenience sample of 20 change leaders and 4 coaches who did not participate in NIATx200. We also consulted with Dr. Sher (Riechmann) Hruska, a developer of the GRSLSS and TSI instruments about our modified scales. Instruments were then revised based on their input.

### Measures

#### Teaching style

The Quality Improvement Coach Teaching Style Inventory (QICTSI) consists of 40 questions, eight questions for each of the five teaching styles. Coach responses to the QICTSI assessed their attitudes and behaviors about coaching in a QIC. The QICTSI included demographic questions related to education; years of experience providing quality improvement coaching and the number of organizations coached in the past 5 years. Coaches also provided suggestions for improvements to the language or wording within the QICTSI.

#### Learning style

The modified Quality Improvement Learning Style Survey (QILSS) is comprised of 60 questions, ten questions for each of the six learning styles. Respondents (change leaders and executive sponsors) utilized the QILSS to their rate attitudes, behaviors and preferences regarding learning styles within a QIC. Respondents also answered demographic questions and could provide suggestions on how to improve the language or wording of the QILSS.

Additional file 2 contains the Quality Improvement Coach Teaching Style Inventory and the Quality Improvement Learning Style Survey.

### Sample size

The executive sponsor and change leader from each of the 201 participating clinics coach were invited to participate. Executive sponsors and change leaders who fulfilled the role for more than one NIATx200 clinic received only one invitation. Eighteen NIATx coaches provided direct coaching or facilitated interest circle and learning sessions.

### Sampling strategy and survey administration

Each participating provider in NIATx200 identified a change leader and executive sponsor. Randomization occurred at the organizational level and did not involve randomization of these individuals. The coaches who served as a NIATx coach or change leader in the initial NIATx provider cohort were selected and recruited to participate in NIATx200 study and then assigned to coach organizations based on geographic proximity and within one of the two intervention arms with a direct one on one coaching component. As such, the study only bias was in which of these individuals chose to respond to the surveys related to learning and teaching styles.

NIATx 200 change leaders, executive sponsors and coaches constituted the sample frame for this study. Paper and electronic versions of the survey instruments were developed. Each individual participant was invited to complete either the learning style (change leader and executive sponsor) or teaching style (coach) survey. The study used a variety of approaches to contact the coaches, executive sponsors and change leaders including e-mails, phone calls and direct mailings.

### Analytic plan

A principle components analysis (PCA) was used to examine the appropriate weighting of questions within each of the style categories. We assume that the response to each question in a style category is correlated with the subject’s preference for some aspect of that style. PCA aims to uncover the number of underlying aspects of style suggested by the responses and the strength of each question’s correlation with each of those aspects. The usual approach assumes each question is equally correlated with a single underlying preference and, therefore, each question should be given equal weight when scoring responses. The PCA can suggest, alternatively, that the set of questions should be split into two (or more) sub-groupings, that some questions should be dropped (i.e., the weights should be zero), or (with enough observations) how the weights should vary by question within each sub-grouping. In the absence of other research suggesting the presence of different teaching and learning styles, we retained the original groupings of the 60 learning-style questions and the 40 teaching-style questions and applied PCA models to each of the resulting eleven learning/teaching style question groupings. To address small sample size issues, we simulated the PCA results for similarly sized samples where the number of underlying style aspects and correlation of questions to those aspects were known. The simulated PCA results were compared to the actual PCA results to assess whether the observed results were consistent with a simple hypothesis about the underlying correlation structure for the questions. This approach created two graphs for each teaching and learning style component. The first graph calculated the estimated eigenvalues for each eigenvector in principal components analysis (PCA). For the teaching style data, the black lines are eigenvalues from ten simulated sets of 16 subjects and k = 8 questions under the null hypothesis, H_0_: Q(i) = w X + (1-w) Z(i), where w = 30 %, X is a standard normal random variable and Z(i) is an independent standard normal random variable across i = 1,…,k. That is, all questions depend equally on a common lurking value, X, and an independent noise term, Z(i). Under H_0_, the primary eigenvector should place equal weight on each question (i.e., 1/8^1/2^ = 0.354) with a corresponding eigenvalue equal to [kw^2^ + (1-w)^2^]/[w^2^ + (1-w)^2^] = 2.09. The theoretical values for the remaining eigenvalues are each equal to (k-2.09)/(k-1) = 0.85. The second graph shows the question weights associated with the primary eigenvector for each of the ten simulated samples, along with the primary eigenvector for the observed results.

In his work, Grasha utilized the test norms from his research in the education field to define “cut-points” for low, moderate and high categories for the individual teaching and learning styles [34]. However, the approach was not articulated. Based on responses from the validation of the revised instruments for this study, we defined the cut-points by calculating the average response for each individual teaching and learning style and then taking ± one standard deviation to determine the different categories. In this instance, style scores between the average ± one standard deviation were considered to be in the moderate category with the high and low categories defined by the scale anchor on the Likert scale and the low or high end of the moderate category respectively.

## Results

### Respondent demographics

**Fig. 1:**
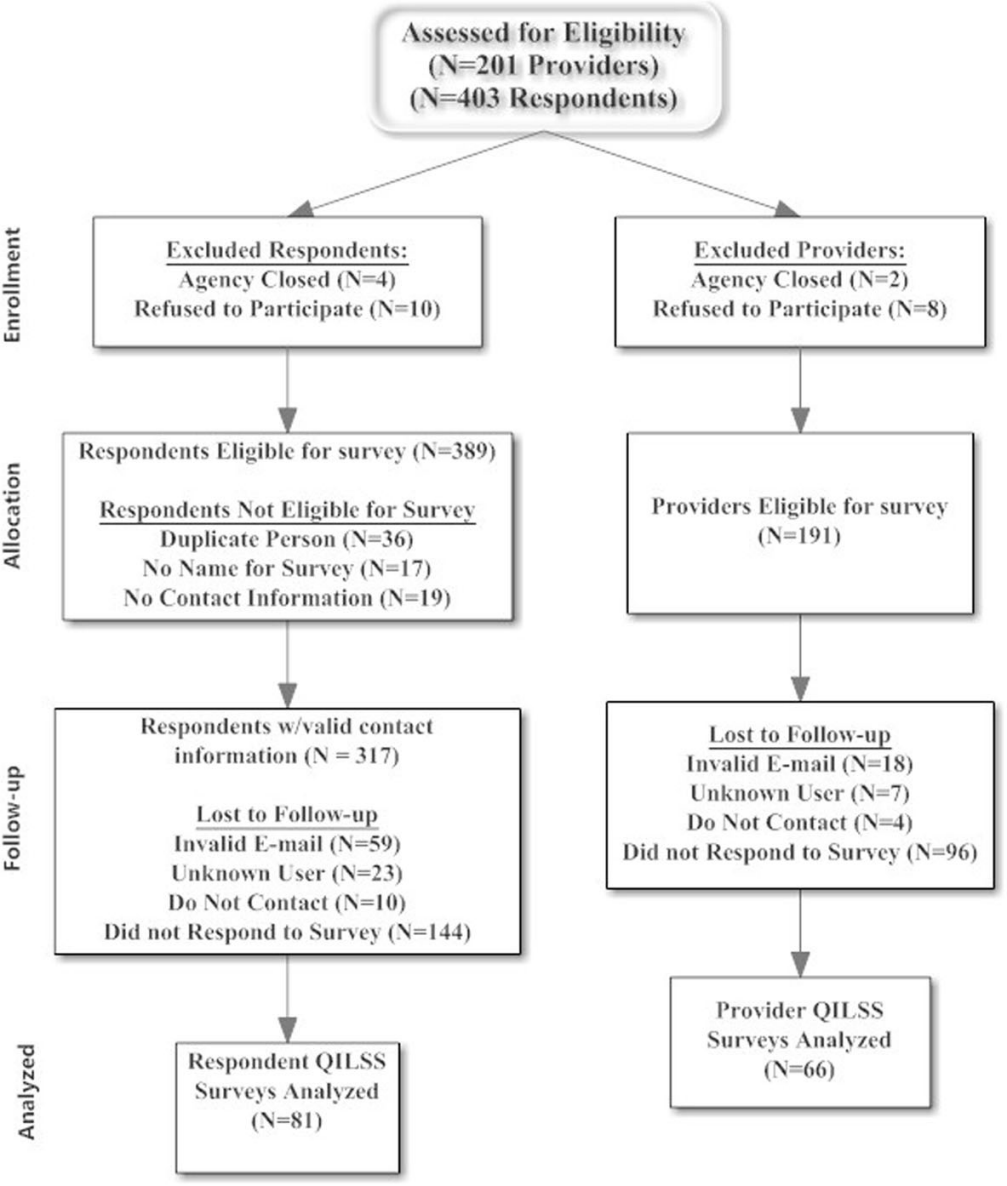
Survey consort diagram by provider and respondents

Responses were received from 17 of the eighteen (94.4 %) coaches in the NIATx200 study. Table 1 shows respondent demographics. Efforts to reach executive sponsors and change leaders were not as successful (Fig. 1 Survey Consort by Provider and Respondents). The final eligible sample size included 191 agencies and 389 respondents. Despite repeated attempts via direct solicitation, e-mail, and phone calls, responses were received from 66 providers (34.6 % of eligible providers). For the individual respondents, 14 change leaders/executive sponsors worked for agencies that had closed or dropped out of the NIATx200 study prior to implementation. 72 individuals were excluded because they represented multiple agencies or had no valid contact information. For the remaining contactable sample (*n* = 317), 80 (25.2 %) change leaders or executive sponsors responded. Three surveys were returned blank and excluded from the final analysis. The difference between the numbers of agencies and respondents in Fig. 1 indicates that two surveys were received (change leader and executive sponsor) from 15 agencies. Table 1 shows the demographics of the coaches’ as well executive sponsors and change leaders. The majorities of respondents were female and had a master’s degree or professional degree. At the time of the study, coach experience was equally distributed and the coaches had worked with an average of 30 providers over the past 5 years. Approximately 55 % of the respondents were change leaders in their organization. The average tenure for the change leaders and executive sponsors was 16 years with their organization and 23 years in the behavioral health field.

**Table 1:** Response rate and respondent demographics

	Coaches (*N* = 17)	Executive sponsor and change leaders (*N* = 81)
Gender^1^	% (*N*)	% (*N*)
Male	41.2 (7)	34.6 (28)
Female	58.8 (10)	63.0 (51)
Refused/No Response		2.5 (2)
Educational Level	% (*N*)	% (*N*)
Some College Courses		3.7 (3)
Some College or Two Year Associate Degree		16.0 (13)
Bachelor’s Degree	5.9 (1)	11.1 (9)
Master’s Degree	76.5 (13)	66.7 (54)
Professional or Doctorate Degree	17.6 (3)	3.7 (3)
Refused/No Response		2.5 (2)
Years of Experience (Coaching)	% (*N*)	% (*N*)
Less than or equal to 5 Years	29.4 (5)	
6 to 9 Years	29.4 (5)	
10 Years or Greater	35.3 (6)	
Refused/No Response	5.9 (1)	
Role in NIATx200^2^	% (*N*)	% (*N*)
Executive Sponsor		45.7 (37)
Change Leader		54.3 (44)
Experience in Years^3^	Mean (SD)	Mean (SD)
Agencies Coached in Past 5 Years	29.6 (24.2)	
Organization		15.5 (7.8)
Behavioral Health Field		23.4 (9.2)

1. The majority of the coaches (82.4 %) and of the executive sponsors/change leader (92.6 %) were Caucasian

2. Four respondents had dual roles (executive director or change leader) for the same provider

3. Three coaches did not respond about agencies coached and two respondents did not answer about tenure

### Principal components analysis

The results from the principal components versus simulated results confirmed the teaching or learning style components to explain the results, we will use the questions related to the *Expert Teaching Style* as an example. The first graph (see Additional file 3) indicates that the primary eigenvectors of the ten simulated samples are somewhat greater 2.09 and the remaining eigenvalues do not appear constant at 0.85. This is because the sample covariances among the simulated questions are subject to estimation error. The simulated non-primary eigenvalues are distributed about 0.85. The PCA routine always sorts the estimated eigenvalues from high to low, giving the appearance of a systematic decrease in explained variance with each additional component. When we anticipate the is sorting effect (i.e., using the ten simulated samples), we see that the pattern of eigenvalues for the actual data (related in this case to the “Expert” domain of questions) is consistent with the null hypothesis that equal weights are appropriate on each question in the domain. In the second graph for the “Expert” domain, the simulated weights are distributed loosely about 0.354 and values for the observed data’s primary eigenvector are not unusual. With only 16 subjects, the observed pattern of Expert question responses is not inconsistent with the assumption that equal weights are appropriate on all questions, i.e., that there is a single lurking effects shared by all of the questions. The conclusion from the analysis for the Expert Teaching style was the presences of a single lurking shared effect, representing 30 % of the observed variation. Additional file 3 contains the complete results of the principal components versus simulated results for the Teaching and Learning styles.

### Coaching styles in NIATx200

Analysis of the Quality Improvement Coach Teaching Style Inventory responses identified the presence of five teaching styles within a QIC. The principal components analysis suggested the exclusion of Question 6 in both the Facilitator and Delegator coaching style (see Additional file 3, pages 4 and 5 respectively). These questions read as follows: “My coaching style encourages a change leader/team to take initiative and responsibility for their learning.” (Facilitator) and “My approach to coaching is similar to a manager of a work group who delegates tasks and responsibilities to subordinates.” (Delegator).

Descriptive statistics for the five coaching (i.e., teaching) styles and categories are shown in Table 2. The score distribution varied within coaching styles and across coaches. For example, the twelve of the seventeen coaches had an above average facilitator score suggesting that coaches in a QIC may prefer to use a facilitation based coaching style. The categorization of high versus low scores was distributed across the coaches with 76.5 % (13 out of 17) being in one of these categories. For example, scores for one coach (C002) placed them in the low range across all five coaching styles. However, the scores for that coach were higher for the facilitator and delegator coaching styles perhaps indicating a preference for using these two styles when coaching in a QIC. Building on the original teaching style definitions, the description and definitions of the resulting coaching styles within a QIC have been expanded and tailored to this setting (see Additional file 4).

**Table 2:** Coaching style statistics within a quality improvement collaborative

	Primary and secondary coaching style scores ^a^							
Coach	Revised delegator	Expert	Revised facilitator	Formal authority	Personal model	Coach intervention assignment ^b^	Primary coaching profile	Secondary coaching profile
C001	*4.86*	*3.63*	*4.86*	*2.63*	*2.88*	C	Facilitator/Delegator	Expert
C002 ^c^	6.00	**5.75**	6.57	**5.25**	**6.50**	CCM	Facilitator/Personal Model	Expert/Formal Authority
C003	6.71	**5.63**	5.00	**4.75**	**4.88**	ICC, CB	Delegator	Expert
C004 ^d^	6.00	4.88	6.57	4.50	4.63	C, LS	Facilitator/Delegator	Expert/Formal Authority/Personal Model
C006	5.14	5.50	6.71	4.50	**6.38**	CB	Facilitator/Personal Model	Expert
C007	5.71	4.63	6.14	3.50	4.75	LS	Facilitator	Delegator
C008	5.29	*3.88*	5.71	4.13	5.00	CB	Facilitator/Delegator	Personal Model
C009 ^d^	5.71	5.00	6.57	**5.25**	4.63	C, ICC	Facilitator/Delegator	Expert/Formal Authority
C010	5.67	**5.71**	6.83	4.43	4.86	CB	Facilitator	Delegator/Expert
C011	5.57	4.75	6.14	4.63	5.00	ICC,CB	Facilitator	Delegator
C012 ^e^	5.29	5.50	6.29	4.38	5.38	CCM, ICC	Facilitator	Delegator/Expert/Personal Model
C013	5.86	*4.00*	5.71	3.63	4.13	C	Facilitator/Delegator	Expert/Formal Authority/Personal Model
C014	5.71	5.50	6.57	**5.25**	5.50	C, LS	Facilitator/Delegator	Expert/Formal Authority/Personal Model
C015 ^e^	5.86	5.25	**7.00**	3.88	**6.13**	CCM, ICC	Facilitator/Personal Model	Delegator/Expert
C016	*5.00*	5.00	6.43	**5.50**	5.63	ICC,CB	Facilitator	Formal Authority/Personal Model
C017	5.57	*4.25*	**6.86**	*3.00*	*2.63*	C	Facilitator	Delegator
C018 ^d^	*5.00*	4.88	5.57	*2.75*	5.00	CCM	Facilitator	Delegator/Expert/Personal Model
Average	5.59	4.92	6.21	4.23	4.93			
St. Dev	0.46	0.67	0.64	0.89	1.04			
Low	1.00–5.12	1.00–4.25	1.00–5.57	1.00–3.34	1.00–3.89			
Medium	5.13–6.04	4.26–5.59	5.58–6.84	3.35–5.11	3.90–5.96			
High	6.05–7.00	5.60–7.00	6.85–7.00	5.12–7.00	5.97–7.00			

^a^ Scores in bold represent coaches in the high range for the associated style while italic scores represent those coaches with a score in the low range

^b^ Intervention Assignment: Coaching (C), Interest Circle (ICC), Learning Session (LS), Combination (CB) and Coaching only but in both the coach and combination interventions (CCM)

^c^ More providers in Washington State were randomized to the combination intervention resulting in one coach providing services to three providers in the coaching and four providers in the combination intervention arms

^d^ Due to the randomization of providers in Oregon and geographic proximity of the providers, coach (C004) provided services primarily to sites in the coaching intervention and one additional provider in the combination intervention. Coach (C009) worked primarily with providers in the combination intervention but provided services to one provider in the coaching intervention

^e^Two coaches (C012 and C015) coached providers in both the coaching and combination interventions due to a coach who was assigned providers in the combination intervention left the study. Their primary intervention arm as initially assigned was the coaching intervention

### Learning styles in a quality improvement collaborative

**Fig. 2:**
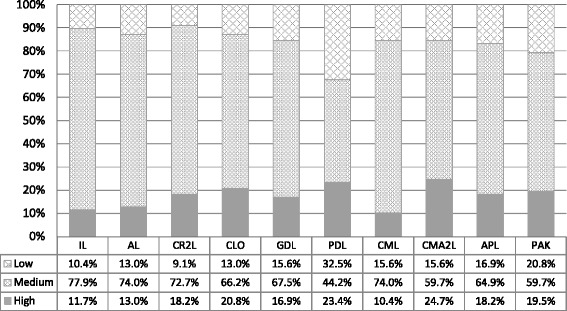
Distribution of QILSS by category

Analysis of the Quality Improvement Learning Style Survey found that the NIATx200 change leaders and executive sponsors preferred one of ten learning styles (Table 3). Questions related to the Independent and Avoidant learning styles as first identified by Grasha loaded onto the same two respective factors as for the NIATx 200 (QIC). For the remaining original four educational based learning styles (Collaborative, Competitive, Dependent and Participatory), the PCA suggests that two learning styles existed within the NIATx 200 QIC for each of these four styles. For example, learners exhibit one of two participatory learning styles reflecting actively participating in learning versus participating “in learning” to acquire knowledge. Table 3 also contains the “cut-points” within each learning style. Figure 2 (Distribution of QILSS by Category) shows the percent of respondents whose score for a particular learning style fell within each “cut-point” category. The average score for three of the ten learning styles, Avoidant Learner, Proximal Dependent Learner and Competitive Leader, were less than 2.50 (one-half of the maximum Likert scale value).

**Table 3:** Learning styles scores within a quality improvement collaborative

QILSS learning style category	Avg.	StDev	Low	Medium	High
Independent Learner (IL)	3.20	0.42	1.00 to 2.78	2.79 to 3.61	3.62 to 5.00
Avoidant Learner (AL)	2.13	0.64	1.00 to 1.49	1.50 to 2.76	2.77 to 5.00
Collaborative Relationship to Learning (CR2L)	4.16	0.70	1.00 to 3.46	3.47 to 4.85	4.86 to 5.00
Collaborative Learning from Others (CLO)	4.18	0.50	1.00 to 3.68	3.69 to 4.67	4.68 to 5.00
Guided Dependent Learner (GDL)	3.43	0.46	1.00 to 2.97	2.98 to 3.88	3.89 to 5.00
Proximal Dependent Learner (PDL)	1.99	0.91	1.00 to 1.08	1.09 to 2.89	2.91 to 5.00
Competitive Leader in Learning (CML)	2.38	0.57	1.00 to 1.81	1.82 to 2.94	2.95 to 5.00
Competitive Approach to Learning (CMA2L)	3.91	0.58	1.00 to 3.33	3.34 to 4.48	4.49 to 5.00
Active Participant in Learning (APL)	3.87	0.55	1.00 to 3.32	3.33 to 4.40	4.41 to 5.00
Participate to Acquire Knowledge (PAK)	3.85	0.47	1.00 to 3.38	3.39 to 4.31	4.32 to 5.00

Table 4 compares the average Quality Improvement Learning Style Survey Learning Style score by respondent role, change leader (*n* = 42) versus executive sponsor (*n* = 35), in NIATx200. Compared to executive sponsors, change leaders’ scores were significantly higher for Active Participant in Learning score, and lower for Avoidant Learner. While not significant, executive sponsors scored higher on Competitive Leader in Learning and Proximal Dependent Learner, and change leaders scored higher on the remaining eight learning styles identified in this study. Similar to the teaching styles, we utilized the original learning style definitions to expand and tailor the description of the resulting learning styles within a QIC (see Additional file 5).

**Table 4:** Change leader versus executive sponsor learning styles

QILSS learning style category	Change leader		Executive sponsor		*P*-Value
	Avg.	SD	Avg.	SD	
Independent Learner (IL)	3.26	0.38	3.13	0.47	0.18
Avoidant Learner (AL)	1.99	0.51	2.30	0.74	0.04
Collaborative Relationship to Learning (CR2L)	4.24	0.65	4.07	0.75	0.30
Collaborative Learning from Others (CLO)	4.28	0.44	4.06	0.54	0.06
Guided Dependent Learner (GDL)	3.52	0.48	3.33	0.42	0.07
Proximal Dependent Learner (PDL)	1.95	0.85	2.03	0.98	0.72
Competitive Leader in Learning (CML)	2.38	0.59	2.38	0.56	0.96
Competitive Approach to Learning (CMA2L)	3.98	0.60	3.83	0.54	0.27
Active Participant in Learning (APL)	3.98	0.50	3.73	0.57	0.04
Participate to Acquire Knowledge (PAK)	3.94	0.38	3.73	0.55	0.05

We received responses to the Quality Improvement Learning Style Survey from the change leader and executive sponsor from 15 different providers. An exploratory analysis (Table 5) suggests that the learning profiles for individuals within an organization may be identical or may differ yet have some of the same learning styles. In seven out of eight instances when the learning profiles are the same, it indicates that the change leader and executive sponsor prefer either a Collaborative/Competitive or Collaborative/Participatory approach to learning and knowledge acquisition. In the final case, both individuals have an Independent Learning profile. In the instances when the profiles differ, a similarity still exists in that the learning profile for each individual (change leader or executive sponsor) has a collaborative component. It may be an indication that the individuals prefer to learn new knowledge when they are able to actively participate with other individuals in the overall learning experience.

**Table 5:** Comparison of learning profiles within the same organization

	Learning profile	
Organization	Change leader	Executive sponsor
Org001	Collaborative/Competitive	Collaborative/Participatory
**Org002**	**Collaborative/Competitive**	**Collaborative/Competitive**
**Org 003**	**Collaborative/Competitive**	**Collaborative/Competitive**
Org004	Collaborative/Participatory	Collaborative
**Org005**	**Collaborative/Competitive**	**Collaborative/Competitive**
**Org006**	**Collaborative/Participatory**	**Collaborative/Participatory**
**Org007**	**Collaborative/Competitive**	**Collaborative/Competitive**
**Org008**	**Collaborative/Participatory**	**Collaborative/Participatory**
Org009	Collaborative/Participatory	Collaborative/Competitive
**Org010**	**Collaborative/Participatory**	**Collaborative/Participatory**
Org011	Collaborative/Participatory	Collaborative
Org012	Collaborative/Dependent	Collaborative/Competitive
Org013	Collaborative/Participatory	Collaborative
Org014	Collaborative/Participatory	Collaborative
**Org015**	**Independent**	**Independent**

Bold rows indicate where the learning style of the change leader and executive sponsor are the same

### Determination of learning and teaching profiles

The educational research suggests that individuals exhibit a preferred combination or group of teaching or learning styles. The PCA results were analyzed to determine if similar combinations exists within a QIC. We visually examined individual learning style score distribution as compared to the average across all change leaders and executive sponsors to determine if a preferred learning style existed for each of these individuals. The results suggested the presence of six learning profiles for the executive sponsors and change leaders within a QIC: Collaborative/Competitive (*N* = 28, 36.4 %); Collaborative/Participatory (*N* = 19, 24.7 %); Collaborative only (*N* = 17, 22.1 %); Collaborative/Dependent (*N* = 6, 7.8 %); Independent (*N* = 3, 5.2 %); and Avoidant/Dependent (*N* = 3, 3.9 %).

A similar approach was utilized to determine if a preferred coaching profile existed for each coach. The results suggested the presence of four primary coaching profiles: Facilitator (*N* = 7, 41.2 %), Facilitator/Delegator (*N* = 6, 35.3 %), Facilitator/Personal Model (*N* = 3, 17.6 %) and Delegator (*N* = 1, 5.9 %). The review of the coaching styles also indicated the presence of secondary coaching profiles. Three coaches (C004, C013 and C014) had a secondary coaching profile of Expert/Formal Authority/Personal Model; another three coaches (C007, C011, and C017) also relied on the Delegator coaching style; and three other coaches (C001, C003 and C006) supported their primary coaching profile through the use of an Expert secondary coaching profile. Six other coaches relied on either the Delegator/Expert (C010, C015); Delegator/Expert/Personal Model (C012, C018) or the Expert/Formal Authority (C009, C002) as their secondary coaching style. The final two coaches (C008, C016) utilized a Personal Model or Formal Authority/Personal Model as their secondary profile. The education literature which our results supported did not suggest that individual learners utilize a secondary approach to learning.

In assigning coaches within the NIATx200 intervention, they either provided only coaching services (*N* = 8) or served as faculty for the interest circle calls or the learning session in addition to their coaching responsibilities (*N* = 8). One coach was faculty for the learning sessions and did not provide direct coaching to providers. Table 2 provides details of these assignments. Using this assignment, we examined the distribution of coaching profiles by intervention arm. The results (not shown) indicated that coaches who provided only coaching services versus fulfilled other roles in the study (i.e., faculty) were equally likely to have either a Facilitator (*N* = 3 per group) or a Facilitator/Delegator (*N* = 3 per group) primary coaching profile. The other coach with a Facilitator profile served as faculty for the learning session. For the three coaches with a primary coaching profile of Facilitator/Personal Model, two provided only coaching services. Only one coach had a Delegator primary profile and they provided coaching services as well as served as a faculty member for the interest circle calls.

## Discussion

Our research was one of the first to our knowledge to assess individual teaching and learning styles within a QIC. We found that the Grasha-Riechmann Student Learning Style Survey and Teaching Style Inventory could successfully be adapted to assess coach teaching style and individual learning styles within a QIC.

The Grasha Riechmann model addressed individual teaching and learning styles and provided evidence for how teachers could leverage and match their teaching approach with different individual learning styles [34]. The structure of the model allows researchers to account for how individual preferences, prior experiences, curriculum design or the learning environment may create learning preferences, approaches or styles that vary from situation to situation [35]. Our study supports this notion.

Findings suggest that learners in a quality improvement collaborative (QIC) utilize multiple learning styles within a broader learning profile. The use of multiple learning styles differs from a recent study indicating a preference for a single learning style [46]. Nine out of 10 learners in NIATx200 preferred one of four learning profiles that included a collaborative learning style. As such, they expect that the structure of the QIC will foster and create co-learning or other opportunities that promote team participation [46–48]. Collaborative learning is only one component of the learning profiles identified in this study. Other profiles include either a dependent or an independent learning style. A profile for a dependent learner appears to indicate a preference to rely more on the coach to guide them through their knowledge acquisition similar to how a tutor would work one-on-one with their student. In contrast, a profile with an independent approach to learning in a QIC appears to represent a combination of learning styles that have been identified as reflective observation, abstract conceptualization and active participation [49] or as one who prefers to reflect on the information they are learning as a part of the process to independent learning [50]. Only three individuals had an independent learning profile in our study. For these persons, they are engaged in a learning process by which they are taking the information learned within the quality improvement collaborative (i.e., the environment) to create their own knowledge about how to best implement change in their organization [49]. Approximately, 35 % of the learning profiles included a competitive learning style. However, NIATx200 did not promote active “competition” among the participating providers and it is unclear why these individuals felt a need to be competitive within the learning collaborative. The idea of profile that includes an avoidant learning style within a QIC setting is perplexing especially when coupled with a perceived dependency on the “collaborative” to support an individuals’ knowledge acquisition. Further understanding of learning styles of individuals who have participated in a QIC is needed to better understand the nature of both the independent and avoidant learner.

Although the sample is small, individuals from the same organization may have similar or different learning profiles. In an education setting, a teacher may have some students who prefer to learn didactically and others who are visual or hands-on learners. In these instances, a good teacher will modify their approach to help all students learn the content. Given that individuals from the same organization learn differently, the inherent challenge for developers of a QIC is to determine how to integrate the different learning profiles to promote active learning and knowledge acquisition. Further research is needed to better understand how this phenomenon plays out in a QIC.

This study is the first, to our knowledge, to assess the preferred teaching style of external coaches in a QIC. The results, based on the available sample, indicated that only the structure of the Delegator and Facilitator styles of NIATx 200 coaches differed significantly from other research on teaching styles (Grasha). The findings suggest seven of the seventeen the coaches had a primary teaching profile that comprised only of facilitation teaching style (*N* = 7). In the educational environment, Grasha defined that facilitation focused on the personal nature of the teacher and student interactions in order to establish the students as independent learners [34]. Within the NIATx200 study, these facilitation skills often resulted in the coaches offering guidance and direction to the change leader/change team to help develop the capacity for independent action, initiative and responsibility. Godfrey and colleagues [51] identified this “helping” approach to coaching to be an essential component in a QIC. For the remaining nine coaches, all except for one had a coaching profile that included the facilitator teaching style. These coaches integrated this style with skills from either the Personal Model (*N* = 3) or the Delegator (*N* = 6) teaching styles. These profiles support the concept that “teachers” utilize multiple styles when delivering content within a learning environment [34]. Only one coach did not use the facilitation in their interactions and instead relied more on the Delegator teaching style when working with their providers.

As suggested by Grasha [34], coaches’ teaching profile also included a reliance on secondary coaching styles. The use of a secondary style allows the coach to use different approaches to motivate the change leader and team to strive towards success. For example, a coach whose secondary coaching profile includes Formal Authority would focus on structure by using rules establishing goals to achieve success. Some aspects of a Formal Authority style encompass a “helping” approach to coaching [51]. The findings support the notion that facilitation is context specific and evolves over time suggesting that one approach to coaching does not fit every situation [52, 53]. However, additional research is required across a wider sample of external coaches to better understand and identify the preferred teaching profiles within a QIC.

Response rate is the primary study limitation. The small sample of coaches (*n* = 17) as compared to the educational sample (*n* = 760) may have contributed to the absence of distinguishable different teaching styles in a QIC versus educational setting. Further research is needed to gather more coach responses and re-evaluate teaching styles as well as profiles in a QIC environment. The usable sample of Quality Improvement Learning Style Survey (QILSS) results was limited to 78 executive sponsor or change leader surveys. An increase in the overall response rate (25.2 %) might have resulted in the identification of the use of different learning styles within a QIC. In turn, staff turnover, closed agency and the elapsed time between this research and the NIATx200 study may have also contributed to the low response rate. Conversely, most, if not all, of the coaches are still actively involved in NIATx related projects and as such, their response rate was much higher (94.4 %). The survey structure and question interpretation are two additional study limitations. How a questions’ wording was presented and interpreted by respondents might have changed the findings if a majority read the question in a similar way. Finally, the retrospective assessment of teaching and learning styles may not have captured the preferred styles at the start of NIATx200 and it is possible that the coaches, change leaders and executive sponsors preferred styles may have changed over time.

## Conclusion

The process of engagement in implementation research suggests that the external coach and change leader relationship is important to QIC success. Often, a collaborative includes multiple approaches to teaching skills and tools to participants in efforts to enhance their learning and knowledge acquisition. Adult learning theory suggests that the match between teaching and learning styles is critical for effective practice-based learning outcomes. With the wide spread use of QICs in healthcare, the identification of individual teaching styles prior to the development of a collaborative provides an opportunity to tailor the coaching intervention or the structure and content delivery mechanism to address the match between the coaching and learning profiles of the participants. Efforts to accommodate learning styles of organizational change leaders or change team members could facilitate knowledge acquisition by delivering content through approaches preferred by individuals with a specific learning profile. Given the time and resource investment required to select and train coaches and change leaders, a more tailored QIC might result in greater improvement in patient and organizational outcomes.

## Additional files

Additional file 1: Original Grasha-Riechmann Styles. (DOCX 14 kb) Additional file 2: Teaching and Learning Styles for QI. (PDF 141 kb) Additional file 3: Principal Component Analysis (PCA) Results. (DOCX 173 kb) Additional file 4: QICTS Definitions. (DOCX 15 kb) Additional file 5: QICLS Definitions. (DOCX 14 kb)

## Abbreviations

GRSLSS: Grasha-Riechmann Student Learning Style Survey
NIATx: Network for the Improvement of Addiction Treatment
PCA: Principal Components Analysis
QIC: Quality Improvement Collaborative
QICTSI: Quality Improvement Coach Teaching Style Inventory
QILSS: Quality Improvement Learning Style Survey
TSI: Teaching Style Inventory
