# Supplementary material for: Adaptation of the Grasha Riechman Student Learning Style Survey and Teaching Style Inventory to assess individual teaching and learning styles in a quality improvement collaborative
Source: BMC Med Educ. 2016 Sep 29;16:252. doi: 10.1186/s12909-016-0772-4 (PMC5041280; doi:10.1186/s12909-016-0772-4)
Supplement: Additional file 1: — Original Grasha-Riechmann Styles. (DOCX 14 kb) [file 12909_2016_772_MOESM1_ESM.docx]

**Grasha Riechmann Student Learning Styles: Student Attributes**

Competitive: learn material to perform better than others; believe that they must compete with other students; like to be at the center of attention; and receive rewards for their performance.

Collaborative: learn by sharing of ideas; cooperative; and like working with others in the class.

Avoidant: non-enthusiastic about learning; do not want to attend class; non-participatory; and uninterested and overwhelmed by the classroom environment.

Participatory: enjoy going to class; participate in class activities as much as possible; and complete as much of the required and optional course requirements as possible.

Dependent: learn only what is required; not intellectually curious; see teacher and peers as providing structure and support; and look to teachers for specific guidance.

Independent: think for themselves; confident learners; prefer to work alone on class projects; and tend to only learn the class content that they feel is important.

**Grasha Riechmann Teaching Styles Attributes**

Expert: possess knowledge and expertise needed to teach the class and strives to maintain their status as an expert in the area and is concerned with transferring the knowledge to ensure students are well prepared.

Formal Authority: status with students is derived due to their knowledge and role as a faculty member. As such, they are concerned with providing feedback and establishing learning goals and expectations to provide students with the structure necessary to learn the materials.

Personal Model: believes in teaching by personal example. As such, they establish an exemplar of how to learn by overseeing, guiding and directing students how to accomplish goals and encouragement to observe and emulate their approach to learning.

Facilitator: places emphasis on the personal nature of the teacher and student interactions. As such, they guide students by asking questions, exploring options, suggesting alternatives, and encourage them to make informed choices. The ultimate goal is to foster independent learners.

Delegator: concerned about developing a students’ capacity to function autonomously and as such, they encourage students to work independently on projects or as parts of autonomous teams where they, as the teacher, are available as a resource.

**Grasha Riechmann Teaching and Learning Style Clusters**

| Cluster | Teaching Style Clusters | Learning Styles |
| --- | --- | --- |
| 1 | Primary: Expert/Formal Authority  Secondary: Personal Model/Facilitator/Delegator | Dependent/Participant/Competitive |
| 2 | Primary: Personal Model/Expert/Formal Authority  Secondary: Facilitator/Delegator | Participant/Dependent/Competitive |
| 3 | Primary: Facilitator/Personal Model/Expert  Secondary: Formal Authority/Delegator | Collaborative/Participant/Independent |
| 4 | Primary: Delegator/Facilitator/Expert  Secondary: Formal Authority/Personal Model | Independent/Collaborative/Participant |
