# Supplementary material for: Adaptation of the Grasha Riechman Student Learning Style Survey and Teaching Style Inventory to assess individual teaching and learning styles in a quality improvement collaborative
Source: BMC Med Educ. 2016 Sep 29;16:252. doi: 10.1186/s12909-016-0772-4 (PMC5041280; doi:10.1186/s12909-016-0772-4)
Supplement: Additional file 2: — Teaching and Learning Styles for QI. (PDF 141 kb) [file 12909_2016_772_MOESM2_ESM.pdf]

ID: _________

Quality Improvement Coach Teaching Style Inventory (QICTSI)

The purpose of this survey is to **assess individual coaching styles in a quality improvement collaborative**. The instrument was adapted from the Teaching Styles Inventory developed by Dr. Anthony Grasha[1]. The survey was restructured to capture and identify the coaching styles of individuals who are supporting organizations or providers in a quality improvement collaborative. Within the survey, the phrase

- **Organization** is meant to cover a wide range of different service providers (e.g., acute and behavioral healthcare as well as community service providers).
- **Quality improvement** refers to the NIATx process improvement model

Survey results will help inform future research grants designed to assess teaching and learning styles within a quality improvement collaborative.

Completion of the survey is entirely voluntary. If you chose to complete the survey, please answer each question to the best of your ability. In return, we will provide you with an overview of your teaching styles and if enough responses are received, a comparison of teaching styles will also be provided.

When answering each question, please use the following seven-point scale and **circle the response which best reflects your individual teaching style**.

Strongly Agree (7) Agree (6) Somewhat Agree (5) Neither Disagree or Agree (4) Somewhat Disagree (3) Disagree (2) Strongly Disagree (1)

**Quality Improvement Coach Teaching Style Inventory Question**	SA	A	SWA	N	SWD	D	SD
1. The facts, concepts, and principles associated with quality improvement are the most important skills that providers should acquire.	7	6	5	4	3	2	1
2. I establish high standards for organizations that I coach in quality improvement.	7	6	5	4	3	2	1
3. What I say and do models appropriate ways for providers to think about issues related to the application of quality improvement content.	7	6	5	4	3	2	1
4. My coaching goals and methods address a variety of individual learning styles.	7	6	5	4	3	2	1
5. The organization change leader/team typically work on change projects with little supervision from me.	7	6	5	4	3	2	1
6. It is very important to share my quality improvement knowledge and expertise with the change leader/team.	7	6	5	4	3	2	1

[1] Grasha AF. *Teaching with style: A practical guide to enhancing learning by understanding teaching and learning styles.* Alliance Publishers; 1996.

**Quality Improvement Coach Teaching Style Inventory Question**	SA	A	SWA	N	SWD	D	SD
7. I am willing to give constructive criticism to the change leader/team when necessary.	7	6	5	4	3	2	1
8. The change leader/team are encouraged to follow examples I provide when teaching quality improvement.	7	6	5	4	3	2	1
9. I allocate time to consult with the change leader/team on how to improve their change projects.	7	6	5	4	3	2	1
10. Activities that I use when teaching quality improvement encourage the change leader/team to develop their own ideas about quality improvement.	7	6	5	4	3	2	1
11. What I have to say about a topic is important for a change leader/team to acquire a broader perspective on the issues in the area of quality improvement.	7	6	5	4	3	2	1
12. The change leader/team would describe my standards and expectations as somewhat strict and rigid.	7	6	5	4	3	2	1
13. I typically show a change leader/team how and what to do in order to master the application of quality improvement in their organization.	7	6	5	4	3	2	1
14. During trainings such as a change leader academy or organizational site visit, I employ small group discussions to help change teams develop their ability to think critically about quality improvement and the application of quality improvement in their organization.	7	6	5	4	3	2	1
15. I encourage the change leader/team to implement one or more self-directed learning experiences to bolster their knowledge of quality improvement.	7	6	5	4	3	2	1
16. When my time with a provider ends, I want a change leader/team to leave well prepared for further work in applying quality improvement to address other organizational change initiatives.	7	6	5	4	3	2	1
17. It is my responsibility to define what a change leader/team must learn and how they should learn it.	7	6	5	4	3	2	1
18. I often use examples from my personal experiences often to illustrate points about the material.	7	6	5	4	3	2	1

Quality Improvement Coach Teaching Style Inventory Question	SA	A	SWA	N	SWD	D	SD
19. I usually guide a change leaders' work on their change project by asking questions, exploring options, and suggesting alternative ways to do things.	7	6	5	4	3	2	1
20. As a coach, it is important to develop the ability of a change leader/team to think and work independently.	7	6	5	4	3	2	1
21. Lecturing is a significant part of how I teach quality improvement within an organization.	7	6	5	4	3	2	1
22. I provide very clear guidelines for how I would like to see quality improvement tasks completed.	7	6	5	4	3	2	1
23. I often show a change leader/team how they can use various principles and concepts.	7	6	5	4	3	2	1
24. My coaching style encourages a change leader/team to take initiative and responsibility for their learning.	7	6	5	4	3	2	1
25. I encourage a change leader to take responsibility for teaching part of the quality improvement approach to other change team members.	7	6	5	4	3	2	1
26. My expertise could be used to resolve differences of opinion about quality improvement content issues.	7	6	5	4	3	2	1
27. In my interactions with a change leader/team, I have very specific goals and objectives that I want to accomplish.	7	6	5	4	3	2	1
28. I provide the change leader/team receive frequent verbal and/or written comments on their performance.	7	6	5	4	3	2	1
29. I typically solicit the change leader/team advice about how and what they want to learn during our calls.	7	6	5	4	3	2	1
30. The change leader/team usually set their own pace for completing rapid cycle change projects.	7	6	5	4	3	2	1
31. The change leader/team members might describe me as a "storehouse of knowledge" who dispenses the fact, principles, and concepts they need.	7	6	5	4	3	2	1

**Quality Improvement Coach Teaching Style Inventory Question**	SA	A	SWA	N	SWD	D	SD
32. My expectations for what I want the change leader/team to accomplish are clearly defined.	7	6	5	4	3	2	1
33. As a result of my coaching efforts, many change leaders/change team members eventually begin to have a broader perspective about their approach to quality improvement.	7	6	5	4	3	2	1
34. The change leader/team usually can make choices among activities (i.e., change ideas to implement) in order to achieve the aim of their change project.	7	6	5	4	3	2	1
35. My approach to coaching is similar to a manager of a work group who delegates tasks and responsibilities to subordinates.	7	6	5	4	3	2	1
36. Depending on the venue (e.g., learning session vs. site visit), this quality improvement approach has more material than I have time available to cover it during my interactions with organizational change teams.	7	6	5	4	3	2	1
37. My approach to teaching quality improvement help the change leader/team develop the discipline needed to successfully apply quality improvement in their organization .	7	6	5	4	3	2	1
38. The change leader/team might describe me as a "coach" who works closely with individuals to correct problems in how they think and behave.	7	6	5	4	3	2	1
39. I give the change leader/team a lot of personal support and encouragement to do well when implementing quality improvement in their organization.	7	6	5	4	3	2	1
40. I assume the role of a resource person who is available to the change leader/team whenever they need help	7	6	5	4	3	2	1

Respondent Demographics

41. What is your gender?
    - ☐ Male
    - ☐ Female
    - ☐ Refused

42. What is your ethnicity?
- ☐ Hispanic/Latino
- ☐ Not Hispanic/Latino
- ☐ Refused

43. What is your race?
- ☐ American Indian/Alaska Native
- ☐ Asian
- ☐ Native Hawaiian or Other Pacific Islander
- ☐ Black or African American
- ☐ White
- ☐ More than one race
- ☐ Refused

44. Please indicate the highest education level you have completed.
- ☐ Stopped school before finishing high school
- ☐ High School degree
- ☐ Some College courses
- ☐ Associate or Technical Degree (2 Year College)
- ☐ Bachelor's Degree (4 Year College)
- ☐ Master's Degree
- ☐ Professional or Doctorate Degree

45. How many years of experience do you have in providing quality improvement coaching? ________

46. For approximately how many organizations have you provided quality improvement coaching in the past five years? _________

Quality Improvement Learning Style Survey (QILSS)

The purpose of this survey is to assess **learning styles of individuals who typically fulfill the role of a change leader or executive sponsor in a quality improvement collaborative.** The instrument was adapted from the Grasha Riechmann Student Learning Style Survey developed by Dr. Anthony Grasha[1]. Originally developed to assess the learning style of students in an academic setting, the language in the survey was restructured in an attempt to capture and identify the learning styles of individuals who are change leaders or executive sponsors within their organizations. Within the survey, the word organization is meant to cover a wide range of different service providers (e.g., acute and behavioral healthcare as well as community service providers).

In this survey, the phrase

Quality improvement refers to the NIATx process improvement model

Course about quality improvement refers to a NIATx learning opportunity such as a Learning Session, Interest Circle Call Change Leader Academy, Webinar etc

Quality improvement coach refers to the individual assigned by NIATx who is your organizational coach

Results from completed surveys will help inform future research grants designed to assess teaching and learning styles within a quality improvement collaborative.

Completion of the survey is entirely voluntary. If you chose to complete the survey, please answer each question to the best of your ability. In return, we will provide you with an overview of your individual learning styles and if enough responses are received, a comparison of teaching styles will also be provided.

If the wording for a particular question is unclear, please let us know how it might be clarified to better assess learning styles in a quality improvement collaborative. You will have the opportunity at the end of the survey to offer your thoughts on what you liked and what could be improved.

When answering each question, please use the following five-point scale (5=Strongly Agree, 4=Agree, 3=Neither Agree or Disagree, 2=Disagree and 1=Strongly Disagree and **circle the response which best reflects your individual learning style**.

**Quality Improvement Learning Style Survey Question**	SA	A	N	D	SD
1. When it comes to implementing quality improvement in my organization, I prefer to work by myself.	5	4	3	2	1
2. I often have trouble focusing.	5	4	3	2	1
3. I enjoy working with other change team members to implement quality improvement in our organization.	5	4	3	2	1
4. I want my quality improvement coach to state exactly what they expect from our organization and me.	5	4	3	2	1
5. To do well, it is necessary to compete with others for the attention of my quality improvement coach or the teacher in a course about quality improvement.	5	4	3	2	1
**Quality Improvement Learning Style Survey Question**	SA	A	N	D	SD

[1] Grasha AF. *Teaching with style: A practical guide to enhancing learning by understanding teaching and learning styles.* Alliance Publishers; 1996.

6. When presented with new information or tools, I do whatever is asked of me to learn the content.	5	4	3	2	1
7. My ideas about quality improvement often are as good as those presented in a textbook about quality improvement.	5	4	3	2	1
8. Activities associated with learning and applying quality improvement in my organization are usually not helpful and boring.	5	4	3	2	1
9. I enjoy discussing my ideas about quality improvement with other individuals from within or outside my organization.	5	4	3	2	1
10. I rely on my quality improvement coach to tell me what content about quality improvement is important for me to learn.	5	4	3	2	1
11. It is necessary to compete with others when implementing quality improvement.	5	4	3	2	1
12. Sessions on quality improvement are typically worth attending.	5	4	3	2	1
13. I usually study what is important to me and not always what the quality improvement coach says is important.	5	4	3	2	1
14. I am very seldom excited about material covered in quality improvement courses.	5	4	3	2	1
15. I enjoy hearing what other organizations think about issues raised in quality improvement courses.	5	4	3	2	1
16. I want clear and detailed instructions on how implement change within my organization.	5	4	3	2	1
17. In a quality improvement course, I must compete with others to get my ideas across.	5	4	3	2	1
18. I get more out of going attending a course on quality improvement than by not attending.	5	4	3	2	1
19. I usually learn a lot of the content related to quality improvement on my own.	5	4	3	2	1
20. I don't want to attend most of the quality improvement courses offered to my organization.	5	4	3	2	1
21. Change leaders from different organizations should be encouraged to share more of their ideas about quality improvement and its application with each other.	5	4	3	2	1
22. I complete assignments exactly the way my quality improvement coach tell me to do them.	5	4	3	2	1
23. To be successful in applying quality improvement in my organization, I have to be aggressive.	5	4	3	2	1
24. When applying quality improvement in my organization, it is my responsibility to get as much as I can out of the materials and learning opportunities.	5	4	3	2	1
25. I feel very confident about my ability to learn on my own.	5	4	3	2	1
26. Paying attention during courses on quality improvement is difficult for me to do.	5	4	3	2	1
**Quality Improvement Learning Style Survey Question**	SA	A	N	D	SD

27. I like to work with other change team members when implementing change in our organization.	5	4	3	2	1
28. Trying to decide what to learn or how to apply quality improvement in my organization makes me uncomfortable.	5	4	3	2	1
29. I like to solve problems or answer questions before anybody else can.	5	4	3	2	1
30. Activities presented in courses about quality improvement are interesting.	5	4	3	2	1
31. I like to develop my own ideas about how to apply the course content about quality improvement.	5	4	3	2	1
32. I have given up trying to learn anything from attending a course on quality improvement.	5	4	3	2	1
33. Attendance at a course about quality improvement makes me feel like part of a team where people help each other learn.	5	4	3	2	1
34. It is important for change leaders to be more closely supervised by their quality improvement coach when implementing change within their organization.	5	4	3	2	1
35. To get ahead in a classroom like setting (e.g., a course on quality improvement), it is necessary to be competitive.	5	4	3	2	1
36. I try to participate as much as I can in all aspects of a quality improvement course.	5	4	3	2	1
37. I have my own ideas about how courses about quality improvement should be run.	5	4	3	2	1
38. When it comes to implementing quality improvement in my organization, I work just hard enough to get by.	5	4	3	2	1
39. An important part of participating in a course about quality improvement is learning to get along with other people.	5	4	3	2	1
40. My notes contain almost everything my quality improvement coach said to me during our conversations.	5	4	3	2	1
41. Being one of the best change leaders is very important to me.	5	4	3	2	1
42. I do all assignments in a course about quality improvement well whether or not I think they are interesting.	5	4	3	2	1
43. If I like a topic presented in a course about quality improvement, I try to find out more about topic on my own.	5	4	3	2	1
44. Instead of planning ahead, I typically rush to complete tasks just prior to a call with my quality improvement coach.	5	4	3	2	1
45. Learning about quality improvement is a cooperative effort between the change leader and quality improvement coach.	5	4	3	2	1
46. I prefer to attend courses about quality improvement (QI) that are highly organized.	5	4	3	2	1
47. To stand out in a classroom like setting (e.g., a QI course), I strive to complete assignments before others.	5	4	3	2	1
48. I typically complete assignments before their deadlines.	5	4	3	2	1
**Quality Improvement Learning Style Survey Question**	SA	A	N	D	SD

49. I prefer to work on projects and assignments by myself.	5	4	3	2	1
50. When it comes to applying quality improvement (QI) in my organization, I prefer that the QI coach leave me alone.	5	4	3	2	1
51. I am willing to help other change leaders out when they do not understand aspects of quality improvement.	5	4	3	2	1
52. When it comes to applying quality improvement in my organization, I like to be told exactly what to do.	5	4	3	2	1
53. I like to know how well other change leaders are doing on their efforts to implement organizational change.	5	4	3	2	1
54. When learning new material such as the application of quality improvement, I complete required assignments as well as those that are optional	5	4	3	2	1
55. When I don't understand something, I first try to figure it out for myself.	5	4	3	2	1
56. During face-to-face learning opportunities, I tend to socialize with people sitting next to me.	5	4	3	2	1
57. I enjoy participating in small group activities.	5	4	3	2	1
58. I want my quality improvement coach to be prepared for our regular coach calls.	5	4	3	2	1
59. I want my quality improvement coach to give me more recognition for the good work I do.	5	4	3	2	1
60. In classes such as a quality improvement course that I attend, I often sit toward the front of the room.	5	4	3	2	1

Respondent Demographics

61. What is your gender?
    - ☐ Male
    - ☐ Female
    - ☐ Refused

62. What is your ethnicity?
    - ☐ Hispanic/Latino
    - ☐ Not Hispanic/Latino
    - ☐ Refused

63. What is your race?
    - ☐ American Indian/Alaska Native
    - ☐ Asian
    - ☐ Native Hawaiian or Other Pacific Islander
    - ☐ Black or African American
    - ☐ White
    - ☐ More than one race
    - ☐ Refused

64. Please indicate the highest education level you have completed.
    - ☐ Stopped school before finishing high school
    - ☐ High School degree
    - ☐ Some College courses

- ☐ Associate or Technical Degree (2 Year College)
- ☐ Bachelor's Degree (4 Year College)
- ☐ Master's Degree
- ☐ Professional or Doctorate Degree

65. How long have you worked for this organization? _________
66. How long have you worked in the field of behavioral health? __________
67. What specific suggestions do you have to improve the learning style survey? For example, were there specific questions that could use more clarity?

If you would like to receive a report that provides the results of your teaching style inventory with comparisons to other responses, please clearly print your name and e-mail in the space provided below.

Name: ____

E-mail: ____
