# Supplementary material for: Adaptation of the Grasha Riechman Student Learning Style Survey and Teaching Style Inventory to assess individual teaching and learning styles in a quality improvement collaborative
Source: BMC Med Educ. 2016 Sep 29;16:252. doi: 10.1186/s12909-016-0772-4 (PMC5041280; doi:10.1186/s12909-016-0772-4)
Supplement: Additional file 3: — Principal Component Analysis (PCA) Results. (DOCX 173 kb) [file 12909_2016_772_MOESM3_ESM.docx]

Appendix 1 –Principal Components Analysis (PCA) versus Simulated PCA Results

- Single lurking shared effect, representing 30% of the observed variation

- Single lurking shared effect, representing 50% of the observed variation

- Single lurking shared effect, representing 50% of the observed variation

- Single lurking shared effect, representing 40% of the observed variation; weight on Q6 is lower than expected (candidate for removal)

- Single lurking shared effect, representing 20% of the observed variation; weight on Q6 is lower than expected (candidate for removal)

- Single lurking shared effect, representing 30+% of the observed variation (Note: I only simulated sample with w = 0%, 10%, 20%, 30%, 40%, 50% and 60%. This case might need, say, w = 35% for a better fit.)

- Single lurking shared effect, representing 40+% of the observed variation; weights on Q1 and Q10 are lower than expected.

- Single lurking shared effect, representing 40% of the observed variation; the eigenvalue for the second component is greater than expected – possibly indicating the need to split the domain – i.e., separating Q1 and Q7.

- Single lurking shared effect, representing 30+% of the observed variation; the eigenvalue for the second component is greater than expected – possibly indicating the need to separate Q5.

- Single lurking shared effect, representing 40% of the observed variation; the eigenvalue for the second component is greater than expected – possibly indicating the need to separate Q7 and Q9.

- Single lurking shared effect, representing 40% of the observed variation; the eigenvalue for the second component is greater than expected – possibly indicating the need to separate Q1 and Q3 (and Q10?).
