# Supplementary material for: Adaptation of the Grasha Riechman Student Learning Style Survey and Teaching Style Inventory to assess individual teaching and learning styles in a quality improvement collaborative
Source: BMC Med Educ. 2016 Sep 29;16:252. doi: 10.1186/s12909-016-0772-4 (PMC5041280; doi:10.1186/s12909-016-0772-4)
Supplement: Additional file 4: — QICTS Definitions. (DOCX 15 kb) [file 12909_2016_772_MOESM4_ESM.docx]

Additional File 3 – Description of Quality Improvement Collaborative Teaching Styles

Expert: In this coaching style, you possess knowledge and expertise that the change leader needs to be successful. As a result, they may strive to maintain their status as a quality improvement expert by displaying detailed knowledge and by challenging change leader/change team to enhance their skills and competence with quality improvement tools and techniques. The coach is concerned with transmitting information and insuring that the change leader/team is well prepared to implement change within their organization.

Formal authority: This coaching style places emphasis on the status you possess with the change leader/team that the coach is working with based on their knowledge and role as a quality improvement coach. When coaching, they are concerned with providing positive and negative feedback, establishing learning goals, expectations, and rules of conduct for change leader/change team. The coach wants to make sure that they provide information about the correct, acceptable, and standard ways to use quality improvement tools and techniques. The coach also wants to provide change leader/team with the structure they need to learn.

Personal model: In this coaching style, the coach believes in “teaching by personal example” and establishes a prototype for how the coach would like to think and behave when interacting with change leader/change team. When coaching, they prefer to oversee, guide, and direct the change leader/change team by showing them how to use a quality improvement tool (e.g., flowcharting) and encourage them to observe and then to emulate your approach.

Facilitator: This coaching style emphasizes the personal nature of coach-change leader/team interactions. As a result, the coach guides and directs the change leader/team by encouraging cooperative as well as independent learning activities. They are good at questions, exploring options, suggesting alternatives, and encouraging the change leader/team to make informed choices. The coaches’ overall goal is to develop in the change leader/team the capacity for independent action, initiative, and responsibility. In coaching, they strive to work with change leader/team on projects in a consultative fashion and tries to provide as much direction, support, and encouragement as possible.

Delegator: Within this coaching style, the coach is concerned with developing change leader/change teams capacity to function in an autonomous fashion. As such, the coach is interested in having people become self-directed, self-initiating learners. The change leader/change team works independently on their quality improvement projects. The coach is available at the request of change leader/change team as a consultant and resource person.
