# Supplementary material for: Adaptation of the Grasha Riechman Student Learning Style Survey and Teaching Style Inventory to assess individual teaching and learning styles in a quality improvement collaborative
Source: BMC Med Educ. 2016 Sep 29;16:252. doi: 10.1186/s12909-016-0772-4 (PMC5041280; doi:10.1186/s12909-016-0772-4)
Supplement: Additional file 5: — QICLS Definitions. (DOCX 14 kb) [file 12909_2016_772_MOESM5_ESM.docx]

Additional File 4 – Description of Quality Improvement Collaborative Learning Styles

Collaborative Relationship to Learning (CR2L): Individual relationships in the learning environment are important to the participant and they prefer to work with others to learn how to implement change. As such, they expect that the coach/faculty in the quality improvement collaborative will encourage participants to share ideas, integrate small group learning activities into the curriculum and take steps to ensure that the overall structure of the learning opportunity promotes collaborative learning.

Collaborate to Learn from Others (CLO): Individual prefers to discuss and share ideas with other persons outside of their organization. The preferred learning style for these individuals appears to focus more on a “one-on-one” approach to learning from others participating in the quality improvement collaborative.

Competitive Leader in Learning (CML): Individual exhibits a competitive nature and prefers to stand out and be a leader in the learning environment. As such, they strive to complete assignments before others and it is very important to be “recognized” as one of the best change leaders in a QI collaborative.

Competitive Approach to Learning (CMA2L): Individual believes that it is necessary to take a competitive approach in their learning within a quality improvement collaborative. As such, they may feel a need to be more assertive when learning new material and “compete” for the attention of and recognition from the coach/faculty in the quality improvement collaborative; prefer to seek out answers before others and have a need to know how their performance compares to others in the quality improvement collaborative.

Active Participant in Learning (APL): Individual takes an active role when learning new material and strives to actively complete all assignments including optional assignments by doing whatever is necessary to learn the content. To promote this learning style, these individuals are more likely to sit towards the “front of the class”.

Participate to Acquire Knowledge (PAK): Individual believes that QI learning opportunities are worth attending and strive to participate in and complete all learning assignments no matter how important or non-important they may perceive the activity in order to promote their knowledge acquisition.

Guided Dependent Learner (GDL): These individuals expect that the coach/faculty of the collaborative will use clear and detailed instructions to state exactly what is expected to promote learning and guide the implementation of quality improvement in their organization. The guidance appears to be driven in part by their adversity to making decisions about implementing quality improvement in their organization. As such, these individuals prefer to attend highly organized learning opportunities; take detail notes on interactions with their coach and expect the coach to be prepared for their regular calls.

Proximal Dependent Learner (PDL): Individual learner seems to prefer a very close coaching relationship and seem to prefer to be closely supervised by their coach on how to implement QI in their organization.

Avoidant Learner (AL): Individual may not to believe in the benefits of how quality improvement might be used in their organization and actively avoid completing learning activities. As such, they may not be excited about attending learning opportunities related to quality improvement; may have difficulty paying attention in these courses and may tend to “rush” to complete tasks prior to calls with their quality improvement coach. When working with these types of learners, the coach might need to first recognize their preferred learning style and take “steps” to trying to actively engage these individuals using a secondary learning style as they may be using this learning style in conjunction with other learning styles.

Independent Learner (IL): Individual prefers to learn about new material and implement projects on their own without assistances from others in their organization. As such, they are confident about their ability to learn on their own without a lot of support from their coach and prefer to work independently on projects. These learners will also develop their own ideas about how to best to teach as well as implement different quality improvement materials into courses or within the organization.
